# TAK1-TABs Complex: A Central Signalosome in Inflammatory Responses

**DOI:** 10.3389/fimmu.2020.608976

**Published:** 2021-01-05

**Authors:** Yan-Ran Xu, Cao-Qi Lei

**Affiliations:** Hubei Key Laboratory of Cell Homeostasis, Frontier Science Center for Immunology and Metabolism, State Key Laboratory of Virology, College of Life Sciences, Wuhan University, Wuhan, China

**Keywords:** TAK1, TABs, post-translational modifications, NF-κB, inflammation

## Abstract

Transforming growth factor-β (TGF-β)-activated kinase 1 (TAK1) is a member of the MAPK kinase kinase (MAPKKK) family and has been implicated in the regulation of a wide range of physiological and pathological processes. TAK1 functions through assembling with its binding partners TAK1-binding proteins (TAB1, TAB2, and TAB3) and can be activated by a variety of stimuli such as tumor necrosis factor α (TNFα), interleukin-1β (IL-1β), and toll-like receptor ligands, and they play essential roles in the activation of NF-κB and MAPKs. Numerous studies have demonstrated that post-translational modifications play important roles in properly controlling the activity, stability, and assembly of TAK1-TABs complex according to the indicated cellular environment. This review focuses on the recent advances in TAK1-TABs-mediated signaling and the regulations of TAK1-TABs complex by post-translational modifications.

## Introduction

The transcription factor nuclear factor kappa B (NF-κB) plays central roles in a variety of cellular events, such as immune and inflammatory responses, cell proliferation, autophagy, tissue remodeling, and metabolic regulation (1–4). In resting cells, NF-κB is sequestered in the cytoplasm where it is associated with inhibitory proteins known as IκBs (5–7). In response to various stimuli, including proinflammatory cytokines tumor necrosis factor α (TNFα) and interleukin-1β (IL-1β), lipopolysaccharides (LPS), and the viral or bacterial infections, the IκB proteins are rapidly phosphorylated by upstream IκB kinases (IKKs) (8, 9). These kinases, consisting of catalytic subunits IKKα, IKKβ, and NF-κB essential modulator (NEMO), phosphorylate serine 32 (Ser32) and Ser36 of IκBα, which leads to the polyubiquitination of IκBα by SCF-βTrCP E3 ligase (SKP1-CUL1-F-box ligase containing the F-box protein βTrCP) and subsequent degradation through 26S proteasome (10–12). NF-κB is then liberated and translocated to the nucleus, thereby initiating the transcription of specific target genes (6, 12–14).

Transforming growth factor-β-activated kinase 1 (TAK1), a serine/theronine kinase, was first identified as a member of the MAPK kinase kinase (MAPKKK) family and was originally found to be a mediator of signal transduction in response to TGF-β or bone morphogenetic protein 4 (BMP-4) (15, 16). Over the years, it has been proved that TAK1 is activated by dozens of stimuli and then phosphorylates a series of target proteins, which elicits different signal transduction and cellular responses across different stresses or cell types (17). Genetic experiments have shown that the *Drosophila* dTAK1 functions downstream of the Imd protein and upstream of IKK complex in the Imd pathway, and activates JNK and NF-κB after immune stimulation (18, 19). It has been well characterized that TAK1 is essential for TNF receptor (TNFR)-, IL-1 receptor I (IL-1RI)-, and Toll like receptors (TLRs)-mediated activation of NF-κB and MAPKs (8, 12, 20, 21). In addition, TAK1 plays critical roles in adaptive immunity by mediating T cell and B cell receptor signaling (22–25). In all of these pathways, TAK1 is considered as a key regulator of activation of NF-κB and MAPKs, and it plays vital roles in transmitting the upstream signal from the indicated receptor complex to the downstream signalosomes (23, 26–28).

Conventional ablation of TAK1 results in embryonic lethality because of bone marrow and liver failure in mice (29–31). TAK1-deficiency in mouse embryonic fibroblasts (MEFs) severely impairs TNFR1-, IL-1R-, and TLRs-mediated activation of NF-κB and MAPKs (20). However, TLR4-mediated activation of NF-κB, p38, and JNK, and the production of proinflammatory cytokines are increased in TAK1-deficient neutrophils, suggesting a cell type-specific role for TAK1 in TLR signaling (32).

TAK1 activation requires TAK1-binding protein 1 (TAB1), TAB2, and TAB3. TAB1 is an adaptor protein constitutively associated with N-terminal kinase domain of TAK1 even in the unstimulated cells, while TAB2 and TAB3 bind to the C-terminus of TAK1 through TAK1-binding domain after stimulation (33–36). Although the excessive production of TAB1 increases the kinase activity of TAK1 and acts as an activator of NF-κB signaling *in vitro*, TAB1-dificiency has minor effects on TNFα- and IL-1β-triggered activation of NF-κB and the induction of downstream inflammatory cytokines (20, 33, 37, 38). Interestingly, TAB1-deficiency dramatically impairs phosphorylation of TAK1 at theronine 187 (Thr187) after TNFα and IL-1β stimulations (39). In contrast, several studies proved that TAB2 and its homolog TAB3 play redundant roles in TAK1 activation (40, 41). Unlike TAB1, TAB2 and TAB3 do not activate TAK1 *in vitro*. Double deficiency of TAB2 and TAB3 has minor effects on TAK1 activation and production of downstream inflammatory cytokines at the early phase after IL-1β stimulation. But in late phase, IL-1β-triggered TAK1 activation and transcription of downstream genes were markedly declined in TAB2- and TAB3-double deficient cells, suggesting that TAB2 and TAB3 are not required in the early TAK1 activation but are essential for sustained TAK1 activation (20, 37, 40, 42).

The TAK1-TABs complex phosphorylates IKKβ at Ser177 and Ser181, which is required for the activation of NF-κB signaling (43–46). In addition, TAK1-TABs complex is also critical for the activation of MAPKs (20, 47). Several reports have demonstrated that the TAK1-TABs complex plays important roles in innate immune and inflammatory responses. It is also emerging that this complex controls a large amount of physiological and pathological processes (21, 48–51). Although the TAK1-TABs complex has been widely studied, the roles of the individual binding proteins and the molecular mechanisms responsible for their activation in different cell types remains to be addressed. In this review, we will summarize the latest advances in the understanding of TAK1-TABs-mediated signaling transduction and the regulation of their activities by post-translational modifications (PTMs).

## The TAK1-TABs-Mediated Signaling Pathway

**Figure 1:**
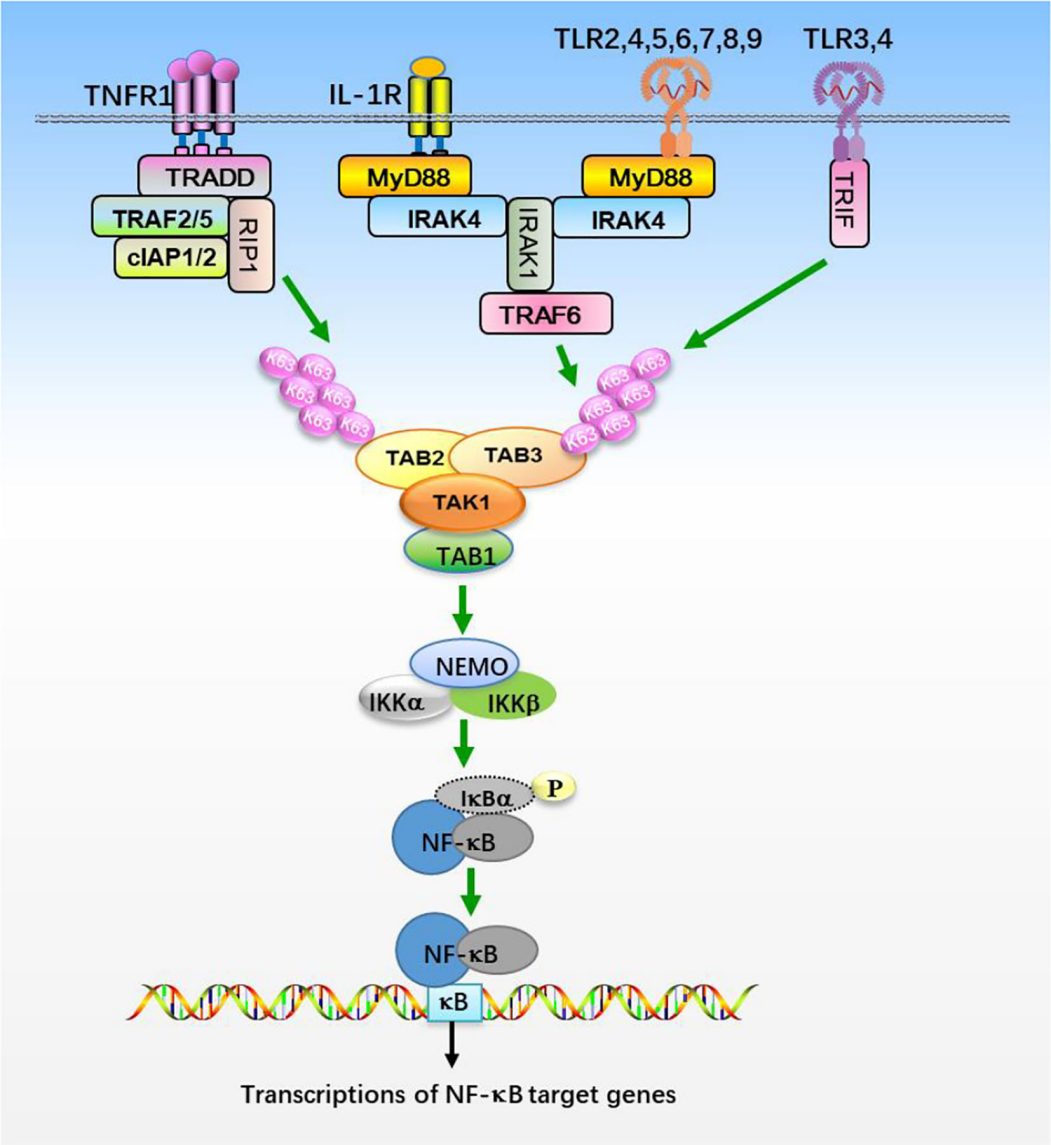
The NF-κB signaling pathway. Stimulation of TNFR1, IL-1R, and TLRs with their ligands promotes recruitments of the indicated adaptor proteins and E3 ligases, which catalyzes the synthesis of K63-linked polyubiquitin chains that preferentially bind to TAB2 and TAB3 subunits, resulting in the assembly and activation of TAK1-TABs complex. TAK1-TABs then phosphorylates IKKs, which activates transcription factor NF-κB and elicits the transcription of downstream genes.

TAK1 is activated by proinflammatory cytokines, such as TNFα and IL-1β, and mediates activation of nuclear factor κB (NF-κB), c-Jun N-terminal kinase (JNK), and p38 (19, 23, 52). IL-1β is an effective inflammatory cytokine that activates NF-κB and other signaling pathways, which are essential for an effective immune response against microbial infections (53, 54). IL-1β exerts its biological function through the binding to interleukin-1 receptor type 1 (IL-1R1), which contains intracellular Toll and IL1 receptor (TIR) domains (**Figure 1**). After binding to IL-1β, IL-1R1 forms hetero-oligomers with IL-1R accessory protein (IL-1RAcP) and recruits the adaptor protein myeloid differentiation primary response protein 88 (MyD88). MyD88 in turn recruits IL-1 receptor-associated kinase 4 (IRAK4) and IRAK1. IRAK4 phosphorylates IRAK1 and releases IRAK1 into the cytoplasm to form signalosome with E3 ligase TRAF6 and induce oligomerization of TRAF6, which in turn triggers its activation (55–61). Activated TRAF6 then functions with E2 enzymes Ubc13 and Uev1A to catalyze the synthesis of unanchored Lys-63 (K63)-linked polyubiquitin chains, which preferentially bind to highly conserved zinc finger (ZnF) domain of TAB2 and TAB3, leading to the oligomerization and autophosphorylation of TAK1 at Thr184 and Thr187 (28, 46, 62–66). TAK1 then phosphorylates IKKs and MAPKs, leading to the activation of NF-κB and AP-1. Interestingly, TRAF6 is also ubiquitinated in the presence of Ubc5 but not Ubc13-Uev1A, which specifically activates IKKs by directly binding to NEMO, thereby facilitating IKKs activation by TAK1 (62, 66, 67). TNF signals though two receptors, TNFR1 and TNFR2 (**Figure 1**). The binding of TNF to TNFR triggers recruitment of downstream adaptor TNFR-associated protein with a death domain (TRADD) (68, 69). TRADD further recruits TRAF2, TRAF5, cellular inhibitor of apoptosis protein 1 (cIAP1), cIAP2, and receptor-interacting protein 1 (RIP1) to form a receptor complex, where TRAF2 and TRAF5 catalyze K63-linked polyubiquitination of RIP1 (52, 70, 71). The K63 ubiquitin chains recruit TAB2 and TAB3 and form a signal complex with TAK1, which in turn activates TAK1 and leads to the phosphorylation and activation of IKKs (28, 62, 64, 66). IKKs further activates transcription factor NF-κB and elicits the transcription of downstream genes.

Toll-like receptors (TLRs) are the most well-studied pattern recognition receptors (PRRs) and responsible for recognizing the microbial components and intermediates produced during replication, leading to the activation of TAK1 and initiating immune and inflammatory responses (60, 61). Studies of mice deficient in each TLR have shown that TLRs are distinct in their ligand recognition and usage of intracellular adaptor proteins. TLRs have an ectodomain with leucine-rice repeat (LRR) that mediates recognition of pattern-associated recognition patterns (PAMPs), a transmembrane domain and a cytoplasmic tail with a Toll/interleukin-1 (IL-1) receptor (TIR) domain that initiates signal transduction (**Figure 1**). Individual TLRs differentially recruit adaptors containing the TIR domain, such as MyD88, TRIF (TIR domain-containing adaptor-inducing interferon-β), TIRAP (TIR domain-containing adaptor protein), and TRAM (TRIF related adaptor molecule) (61). Except TLR3 signals through TRIF, most TLRs utilize MyD88 for signaling and ultimate activation of NF-κB and MAPKs (61, 72). After TLRs engagement, MyD88 is recruited to TIR domain of TLRs and forms a complex with IRAK1 and IRAK4, where IRAK1 is autophosphorylated or phosphorylated by other kinases (60, 61, 72). Then IRAK1 is released from MyD88 and interacts with E3 ligases TRAF3 and TRAF6. TRAF6 functions with E2 enzymes, Ubc13 and Uev1A, promotes K63-linked polyubiquitination of target proteins, including TRAF6 itself and NEMO (6, 46, 64, 72). Ubiquitinated TRAF6 subsequently recruits TAK1 and TABs, which then activates the IKK complex and MAPKs pathways, respectively (72). Thus the TAK1-TABs complex is critical for TLRs-mediated signaling.

## Immune Regulation of TAK1-TABs-Mediated Signaling by Post-Translational Modifications

Because TAK1-TABs complex exerts essential roles in diverse signaling pathways in response to a wide range of immune stimulation, the regulation of TAK1-TABs-mediated signal cascades have been extensively investigated. TAK1-TABs complex is heavily and dynamically modulated by different post-translational modifications, including phosphorylation, ubiquitination, methylation, acylation, O-GlcNAcylation, and sumoylation, which play important roles in regulating the activity, stability, as well as assembly of TAK1-TABs complex, fine-turning the inflammatory responses. We next summarize the roles and mechanisms of the different PTMs of TAK1-TABs.

### Phosphorylation-Mediated Regulation of TAK1-TABs Signalosome

**Figure 2:**
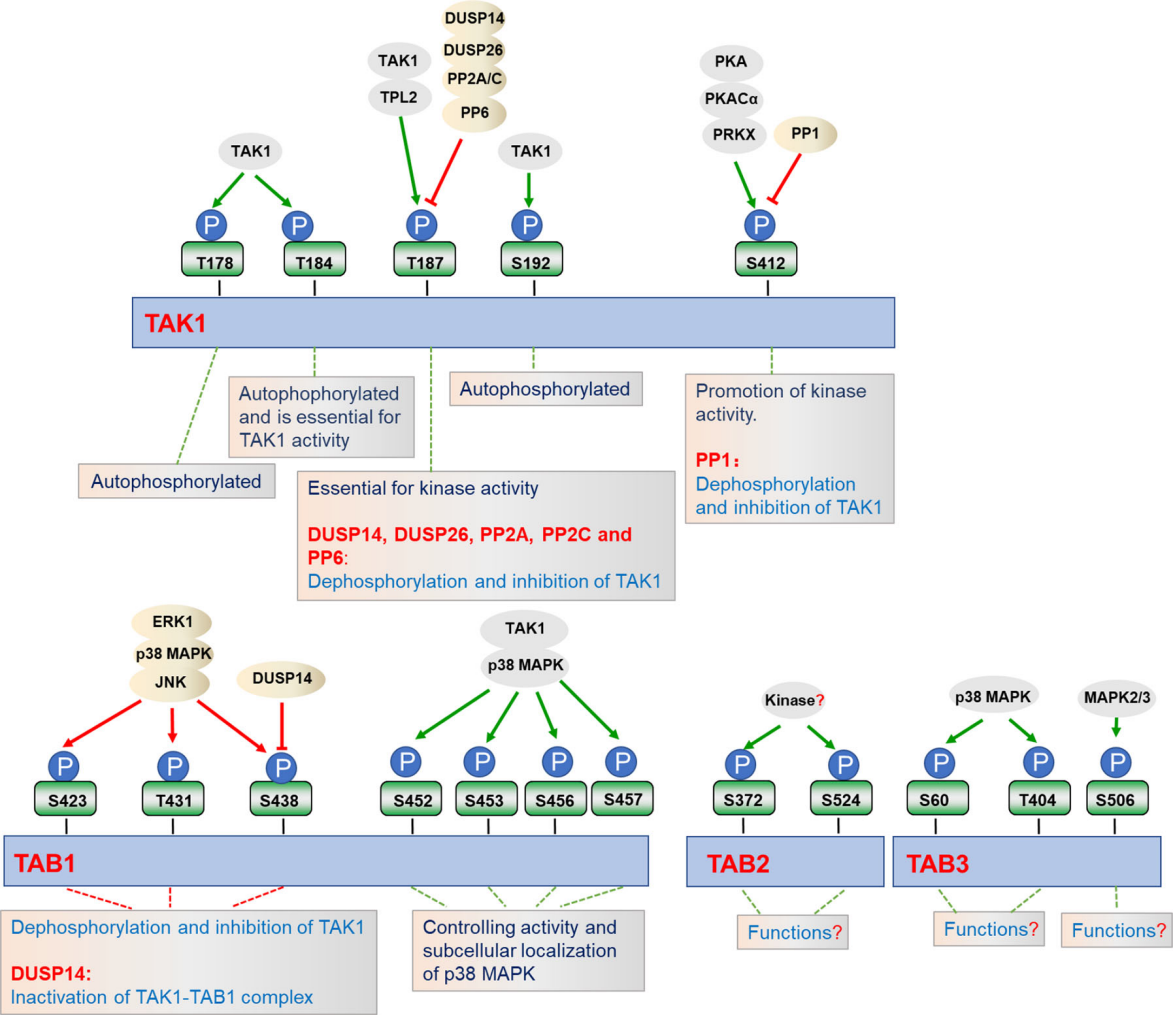
Regulation of TAK1-TABs complex by phosphorylation. TAK1-TABs complex is heavily and dynamically modified with phosphorylation (P) and dephosphorylation by the indicated kinases or phosphatases, which regulates their activity and subcellular localizations.

Phosphorylation and dephosphorylation of critical serine or threonine residues in the activation loop of TAK1 are critical for its kinase activity (**Figure 2**). Four conserved serine and threonine residues, Thr178, Thr184, Thr187, and Ser192, within the kinase activation loop of TAK1 have been reported to be essential for TAK1-mediated activation of NF-κB and AP-1 (23, 38, 63, 73–75). Upon stimulation with inflammatory cytokines, TAK1 undergoes autophosphorylation at Thr187 and Ser192, and then associates with TAB2 and TAB3 (75, 76). Interestingly, deficiency of TAB1 almost completely inhibits TAK1 kinase activity, but TAB1-deficiency has minor effects on phosphorylation of TAK1 at Thr187 and activation of NF-κB stimulated with TNF and IL-1, suggesting that the phosphorylation on Thr187 is dispensable for TAB1 and sufficient for TAK1-mediated activation of NF-κB and MAPKs (23, 37, 39, 77, 78). Prickett et al. have shown that the protein phosphatase subunit known as type 2A phosphatase-interacting protein (TIP) has TAB-qualified properties, so it is also named TAB4. TAB4 directly binds to and enhances the autophosphorylation of TAK1 at Thr178, Thr184, Thr187, and Ser192, which further specifically promotes the phosphorylation of IKKβ and the activation of NF-κB (79). The Thr187 of TAK1 is also phosphorylated by other kinases, such as tumor progression locus 2 (TPL2). TPL2 associates with and phosphorylates TAK1 at Thr187 after IL-17 stimulation, thereby promoting auto-immune neuroinflammation (80). The phosphorylation of TAK1 at Thr187 is critical for TAK1-mediated signal transduction, and a series of phosphatases have been reported to regulate TAK1 activity at different stages following stimulation (75). Dual-specificity phosphatase 14 (DUSP14) associates with and dephosphorylates TAK1 at Thr187, which inhibits TNFα- or IL-1β-induced NF-κB activation. In contrast, DUSP14 enzymatic-inactive mutant DUSP14 (C111S) lost its ability to dephosphorylate TAK1 or inhibit NF-κB activation after simulation (81, 82). Recently, Ye et al. found that another DUSP member, DUSP26, directly binds to TAK1 and mediates dephosphorylation of TAK1, resulting in the alleviation of hepatic steatosis and metabolic disturbance (83). In addition, protein phosphatase 2C (PP2C) and PP6 also dephosphorylate TAK1 at Thr187, but they seem to function on different forms of TAK1. PP2C inhibits TAK1 activity in unstimulated cells, while releasing from TAK1 complex and participating in TAK1 activation after cytokines stimulation. In contrast, PP6 only dephosphorylates TAK1 in an IL-1β-dependent manner, but it has minor effects on TAK1 activity in the resting cells. Moreover, PP2A and PP2C also mediate dephosphorylation of TAK1 at Thr187 after TGF-α and IL-1β stimulation, respectively (84, 85). Yasuhiro et al. found that the phosphorylation at Ser412 is also important for TAK1 activation (86). cAMP-dependent protein kinase A (PKA) mediates the phosphorylation of TAK1 at Ser412, and enhanced activation of NF-κB and MAPKs, which is essential for cAMP/PKA-induced osteoclastic differentiation and cytokine production in precursor cells (76). Dr. Xia’s group showed that the phosphorylation of TAK1 at Ser412 is also mediated by cAMP-dependent protein kinase catalytic subunit α (PKACα) and X-linked protein kinase (PRKX) in TLR- and IL-1R-triggered signaling independent of PKA activation agents (76). It remains to be investigated whether PKACα and PRKX collaborate in promoting the phosphorylation of TAK1 at Ser412. In contrast, PP1 together with its regulatory subunit DNA damage-inducible protein 34 (GADD34) synergistically dephosphorylate TAK1 at Ser412. PP1 and GADD34 associate with TAK1 in unstimulated cells, while disassociating immediately from TAK1 after ligand stimulation, and then quickly interacting with TAK1 again. The cycling of association and disassociation of TAK1 with PP1 thus dynamically controls the activation of IL-1R/TLR signaling and protects the host from excessive inflammatory immune responses (87).

In addition to the modifications of TAK1, TAK1-binding proteins TAB1, TAB2, and TAB3 are also phosphorylated in response to the different stimulation. It has been reported that the four serine residues of TAB1, including Ser452, Ser453, Ser456, and Ser457, can be phosphorylated by TAK1 as well as by p38 MAPK in response to IL-1α, anisemycin, and sorbitol. Interestingly, the phosphorylation of TAB1 at these four serine residues inhibits TAB1-dependent phosphorylation of p38 MAPK, but it does not affect the activation of TAK1, suggesting these phosphorylation may have unknown roles in stimulus-specific activation of TAK1-TAB1-mediated signaling (88). Through mass spectrometry and phosphosite-specific antibodies, TAB1 is also shown to be phosphorylated on S423, T431, and S438 by ERK1, p38 MAPK, or JNK, which in turn dephosphorylates and inactivates TAK1 (39, 42). Unlike TAB1, IL-1β-induced phosphorylation of TAB2 at Ser372 and Ser524 are dispensable for p38 MAPK or JNK. In contrast, Ser60 and Thr404 of TAB3 are phosphorylated directly by p38 MAPK, where Ser506 is phosphorylated by mitogen activating protein kinase (MAPK2) and MAPK3, protein kinases which are activated by p38 MAPK (39). Moreover, serine/threonine phosphatases PP2C-, PP6-, or calcineurin-mediated dephosphorylation of TABs may cause their inactivation (84, 85, 89). In addition to their function at TAK1, DUSP14 also directly interacts with and dephosphorylates TAB1 at Ser438, leading to the inactivation of TAK1-TAB1 complex in T cells, which negatively regulates TCR signaling and immune responses (81). IL-1α and IL-1β induce phosphorylation of TAB2 at Ser372 and Ser524 or TAB3 at Ser60, Thr404, and Ser506, but the physiological roles of phosphorylation at these sites have yet to be determined (39). Although the phosphorylation and dephosphorylation of TAK1-TABs complex have been extensively studied, the exact dynamic regulation in specific cells and tissues across different stresses are still largely unknown, and potential new kinases and phosphatases function at TAK1-TABs complex need to be identified in future studies.

### Ubiquitination-Mediated Regulation of TAK1-TABs Signalosome

**Figure 3:**
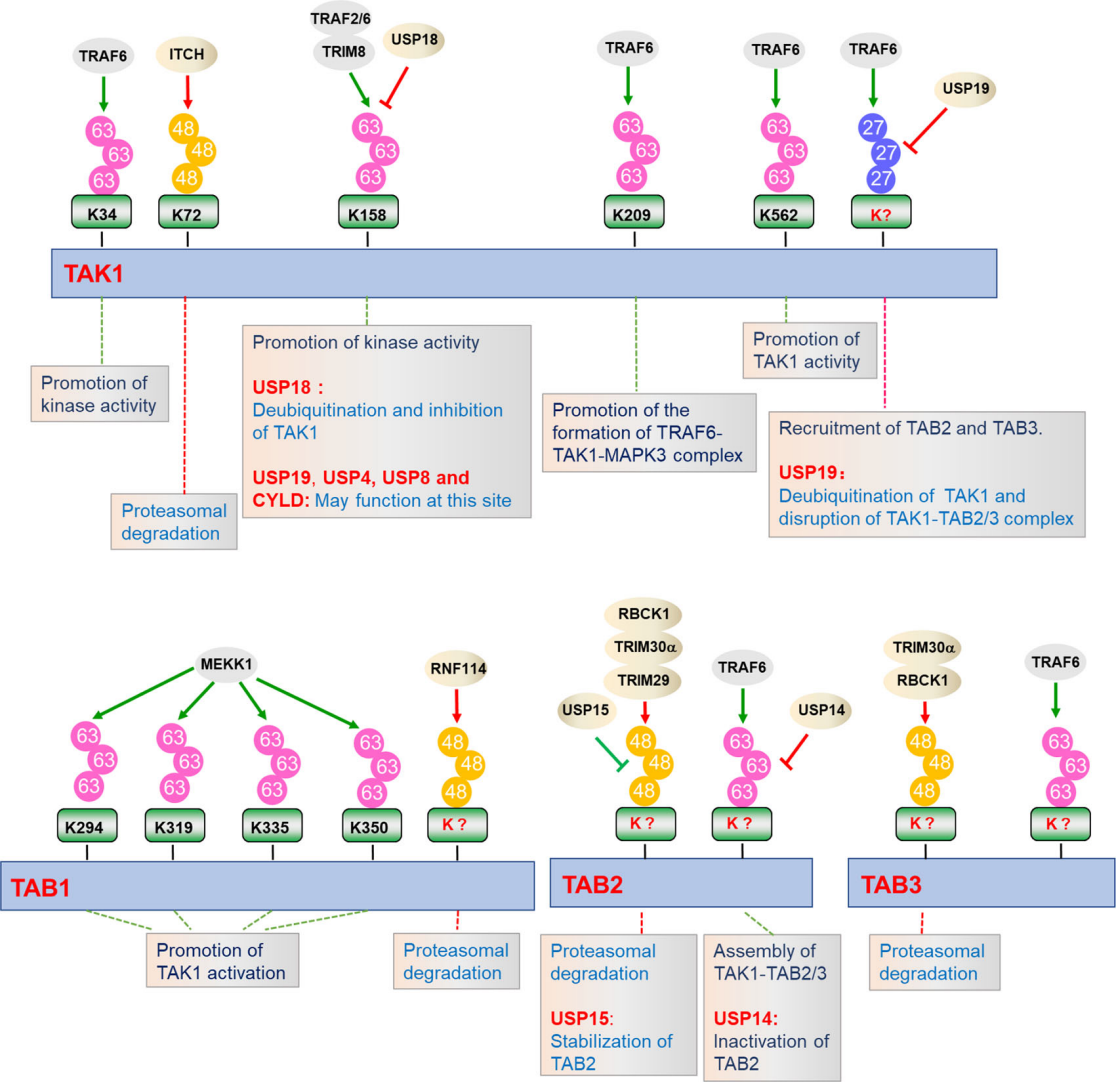
Regulation of TAK1-TABs complex by ubiquitination. TAK1-TABs complex are dynamically modified with K27-, K48-, and K63-linked polyubiquitination by the indicated E3 ubiquitin ligases or deubiquitinating enzymes, which play important roles in regulating the stability, assembly, and activation of TAK1-TABs complex.

Ubiquitin is a 76 amino acids protein containing 7 lysine (K) residues, K6, K11, K27, K29, K33, K48, and K63, any of which can participate in the formation of specific polyubiquitin chains, leading to different destiny of the target proteins (90, 91). It is well established that ubiquitination plays crucial roles in the regulation of TAK1-TABs complex activation (**Figure 3**). Several lysine residues of TAK1, such as Lys34, Lys158, Lys209, and Lys562, have been identified as potential sites for polyubiquitination. Among them, ubiquitination of TAK1 at K158 is required for TAK1-mediated signaling pathways (52, 92, 93). In response to IL-1β, IRAK1 is phosphorylated and released to the cytoplasm with TRAF6, and TRAF6 then collaborates with Ubc13-Uve1A to catalyze the synthesis of K63-linked polyubiquitin chains at Lys158 of TAK1 (52, 94). Our group has found that tripartite motif (TRIM)-containing proteins 8 (TRIM8) serves as a critical regulator of TNFα- and IL-1β-triggered NF-κB activation by mediating K63-linked polyubiquitination of TAK1 at Lys158 (67). Whether TRAF6 and TRIM8 collaborate or are redundant is unclear. In addition, *Helicobacter pylori* (*H. pylori*) cytotoxin-associated gene A (CagA) potentiates TRAF6-mediated polyubiquitination of TAK1 at K158, which is essential for anti-*H. pylori* immune response (95). Chen et al. have further shown that K63-linked polyubiquitination of TAK1 at Lys562 is a prerequisite for the phosphorylation of TAK1 and specifically contributes to the activation of MAPKs, but had minor effect on the formation of TAK1-TABs complex (47). However, the studies of polyubiquitination of TAK1 at Lys34 and Lys209 are still controversial. It has been reported that TRAF6-mediated K63-linked polyubiquitination of TAK1 at Lys34 is important for TAK1 activation in TGF-β signaling pathway (65, 96). When the Lys34 of TAK1 is replaced with arginine (R), the activation of NF-κB and p38 will be impaired after stimulated with LPS, TNFα, IL-1β, or TGF-β, which proves that polyubiquitination of TAK1 at Lys34 is important for the activation of p38 and NF-κB (96, 97). In addition, TRAF6 also mediates the polyubiquitination of TAK1 at Lys209 and promotes the formation of TRAF6-TAK1-MEKK3 signal complex, thereby promoting the continuous activation of NF-κB (65). However, Fan et al. showed that Lys34 and 209 are dispensable for IL-1β-triggered activation of TAK1. Reconstitution of TAK1-deficient MEFs with K209R or K34R mutant of TAK1 could restore the activation of NF-κB and MAPKs to the same level as wild-type TAK1 does after IL-1β stimulation, which suggest that TAK1 activation does not necessarily require polyubiquitination at Lys34 and Lys209 (92, 98). It is possible that Lys34 and Lys209 are differentially required for TAK1 activation in different signaling or different cell types or tissues. Therefore, further research is needed to investigate the physiological functions of K63-linked polyubiquitination of TAK1 at Lys34 and Lys209. In addition, TAK1 is also regulated by other types of polyubiquitination. Recently, our group found that TRAF6 mediates K27-linked polyubiquitination of TAK1, which is a prerequisite for the recruitments of TAB2 and TAB3 to TAK1 to form the TAK1-TABs complex (94).

Polyubiquitination also functions to inhibit TAK1-mediated NF-κB activation by diverse mechanisms. Recently, it has been reported that ITCH (AIP4) inhibits doxycycline (Dox)-induced NF-κB activation by catalyzing K48-linked polyubiquitination of TAK1 at Lys72 residue and promoting TAK1 degradation. Lacking of ITCH results in continuous activation of TAK1 and increased cytokine production in bone marrow-derived macrophages (BMDMs), leading to non-small-cell lung cancer (99, 100).

Emerging evidence indicates that TAB1, TAB2, and TAB3 are modified by a series of E3 ubiquitin ligases, determining their functionality. MEKK1 (also known as MAP3K1) is the only MAP3K that contains PHD motif, which is predicted to function as E3 ligase. During the ES-cell differentiation and tumorigenesis, MEKK1 promotes the ubiquitination of TAB1, which is critical for the activation of MAPKs (101, 102). On the contrary, E3 ubiquitin ligase ITCH directly binds to TAB1 and mediates K48-linked polyubiquitination and degradation of TAB1, which inhibits p38 signaling and skin inflammation in mice (103). RNF114 catalyzes K48-linked polyubiquitination and degradation of TAB1 during maternal to zygotic transition (MZT), which links maternal clearance to early embryo development (104). TAB2 and TAB3 are ubiquitinated by TRAF6 after TNFα and IL-1β stimulation, which facilitates assembly of TAK1-TABs complex (28, 40). TRIM30α is induced by TLR ligands in a NF-κB-dependent manner and targets TAB2 and TAB3 for ubiquitination and degradation, which inhibits TLRs-mediated inflammatory responses (105). Recently, another TRIM family member, TRIM29, has been reported to catalyze the ubiquitination and degradation of TAB2, which impairs IFN-γ production and disordered NK cell functions (106). RBCC (RING-finger, two B-boxes, and α-helical coiled-coil domain) protein interacting with protein kinase C1 (RBCK1) is associated with and ubiquitinates TAB2 and TAB3, which leads to their degradation in a proteasome-dependent manner. Deficiency of RBCK1 potentiates TNFα- and IL-1β-triggered activation of NF-κB (54). In addition, other E3 ligases, including RNF4 and TRIM38, are reported to promote degradation of TAB2 and TAB3 dispensable for their ubiquitin ligase activities (107–109).

Considering the important roles of ubiquitination in TAK1-TABs-mediated signaling, the reverse deconjugation process of ubiquitination would be as equally important (**Figure 3**). Deubiquitination is mediated by a group of proteins called deubiquitinating enzymes (DUBs), which consists of four families. Among them, the ubiquitin-specific proteases (USPs) are widely studied and contribute to the regulation of inflammatory responses (110). Recently, our group found that USP19 is associated with TAK1 in a TNFα- or IL-1β-dependent manner and specifically deconjugates K63- and K27-linked polyubiquitin chains from TAK1, leading to the impairment of TAK1 activity and the disruption of TAK1-TAB2/3 complex. USP19-deficient mice produced higher levels of proinflammatory cytokines and were more susceptible to TNFα- and IL-1β-induced death (94). Unlike USP19, USP18 is an interferon inducible gene (ISG), which is upregulated by TLR ligands in immune cells. USP18 is associated with and removes K63-linked ubiquitin moieties from TAK1-TAB1 complex, thereby negatively regulating TAK1-TABs activity during Th17 differentiation (111, 112). USP18 is expressed at higher levels in Th0, Th1, and Th17 cells than in inducible regulatory T cells, Th2 cell, bone marrow-derived macrophages (BMDMs), and bone marrow-derived dendritic cells (BMDCs). In contrast, USP19 is abundantly expressed in inducible regulatory T cells, Th2, BMDMs, and BMDCs but not in Th0, Th1, and Th17 cells. These observations suggest that USP18 and USP19 deubiquitinate TAK1 in a cell type-specific dependent manner (94, 111). In addition, another ubiquitin-specific protease, USP4, also serves as a critical inhibitor of TNFα-triggered NF-κB activation by removing the K63-linked polyubiquitin moieties from TAK1 (113). Recently, Wang et al. found that p38‐interacting protein (p38IP) inhibits TCR- and LPS-triggered cytokines production. Mechanistically, p38IP dynamically interacts with TAK1 and promotes USP4-dependent deubiquitination of TAK1. Moreover, p38IP also inhibits the unanchored K63-linked ubiquitin chains binding to TAK1 (114). TAK1 activity can also be terminated by deubiquitinase cylindromatosis (Cyld) and E3 ubiquitin ligase Itch complex. In response to TNFα and IL-1β, Cyld expression is upregulated to form a complex with Itch through WW-PPXY motif. Cyld specifically removes K63-linked polyubiquitin moieties from TAK1 and consequent to the inhibition of TAK1 activity. In addition, its partner Itch mediates K48-linked ubiquitination of TAK1, which is then degraded in a proteasome-dependent manner (99, 115). Consistently, Cyld- and Itch-double deficiency leads to chronic and sustained production of inflammatory cytokines by tumor-associated macrophages and aggressive growth of lung carcinoma (99, 100, 115–117). Intermittent hypoxia/reoxygenation (IHR) has been reported to contribute to the activation of NF-κB as well as the induction of inflammatory cytokines. Zhang et al. found that USP8 suppresses the IHR-induced activation of NF-κB and pro-inflammatory cytokines production by removing K63-linked polyubiquitination moieties from TAK1 (118).

In addition to TAK1, TABs are also regulated by deubiquitinating enzymes. Recently, Zhou et al. found that USP15 specifically removes K48-linked polyubiquitin moieties from TAB2 and prevents its lysosome-associated degradation in both DUB activity dependent and independent manner, thus stabilizing TAB2 after TNFα and IL-1β stimulation. On the other hand, USP15 inhibited autophagy cargo receptor 1 (NBR1)-mediated selective autophagic degradation of TAB3. Collectively, USP15 positively regulates TNFα- and IL-1β-triggered NF-κB activation by distinct mechanisms, which reflects the potential nonredundant roles of TAB2 and TAB3 (119). In contrast, USP14 interacts with and removes polyubiquitin moieties from TAB2 and inhibits TAB2 activity. USP14-deficient THP-1 cells showed enhanced activation of NF-κB and production of inflammatory cytokines after LPS stimulation, which suggests that USP14 negatively regulates TLR4-mediated inflammatory responses by deubiquitinating TAB2 (120).

While the ubiquitination and deubiquitination of TAK1-TABs have been extensively studied, and a large number of E3 ubiquitin ligases and deubiquitinating enzymes have been identified, the exact dynamic regulation of ubiquitination and deubiquitination in specific cells in response to different stimuli are still unclear. Therefore, further investigations on these questions and other outstanding questions need to be clarified in future studies.

### Unconventional PTMs-Mediated Regulation of TAK1-TAB Signalosome

**Figure 4:**
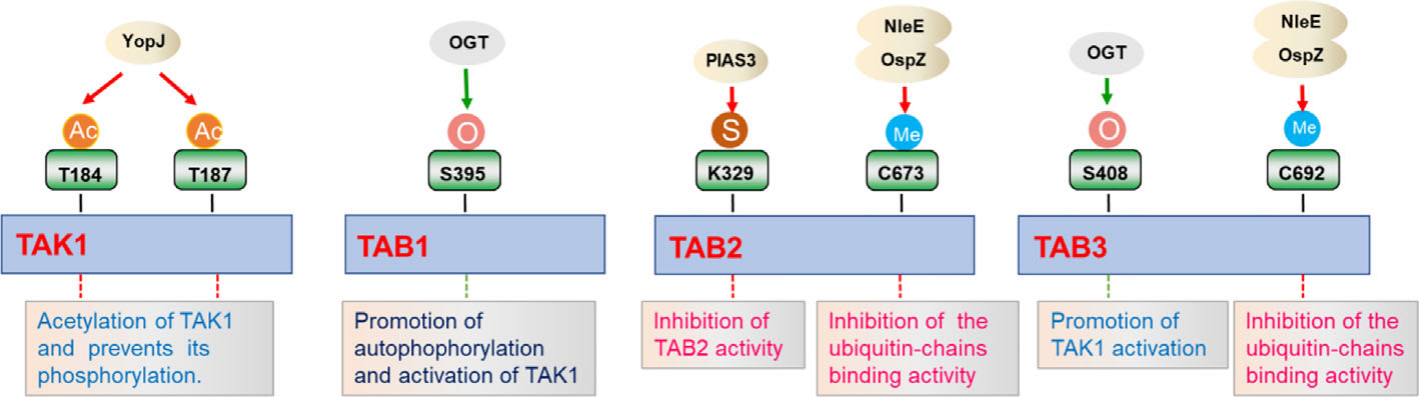
Regulation of TAK1-TABs complex by different post-translational modifications. TAK1-TABs complex is modified by various post-translational modifications including acylation (Ac), methylation (Me), sumoylation (S), and O-GlcNAcylation (O), which regulates the activation and the assembly of the complex in response to different stimulation.

In addition to the more extensively characterized phosphorylation and ubiquitination, TAK1-TABs are also modified by other post-translational modifications, including methylation, O-GlcNAcylation, and sumoylation (**Figure 4**).

Protein methylation is a post-translational modification that transfers a methyl group from the donor S-adenosyl-L-methionine to cytosine in CpG dinucleotides (121–123). Initially, protein methylation was first identified as a modification that regulates chromatin remodeling and gene transcription (121). Up to now, methylation of Lysine or Argine residues on non-histone proteins is an emerging field, displaying important roles in the regulation of signaling for diverse array of pathways (121). NleE, a bacterial type III secreted effector, harbored unprecedented S-adenosyl-L-methionine-dependent methyltransferase activity. After *Escherichia coli* infection, NleE is released into the cytoplasm and specifically methylates TAB2 at Cys673 and TAB3 at Cys692 in their NZF domain, which diminishes their ubiquitin binding capacity and consequent to the inhibition of inflammatory cytokines expression (121, 124, 125). Similar results are also observed for NleE homolog OspZ from *Shigella flexneri*, which reduces IL-8 transcription by methylating TAB2 and TAB3 (121, 126). In addition to methylation, protein acetylation is also one of the common post-translational modifications in eukaryotes. Bacterial effectors from *Yersinia* can act as acetyltransferases to mediate acetylation at the phosphorylation sites of MKK6, IKKα, and IKKβ, thereby inhibiting their phosphorylation and activation. YopJ is one of six effector proteins (Yops) injected into the host cell cytoplasm through type III secretion system during *Yersinia pestis* infection, and inhibits mammalian NF-κB and MAPKs signaling pathways (127, 128). Recent studies indicate that YopJ acts as a serine/threonine acetyltransferase that targets dTAK1. Acetylation of key serine/threonine residues in the activation loop of *Drosophila* TAK1 prevents its phosphorylation and subsequent inhibition of kinase activation. Furthermore, YopJ also inhibits TAK1 activity in mammalian cells. YopJ prevents phosphorylation of TAK1 by acetylating TAK1 at Thr184 and Thr187, inhibiting autophosphorylation of this kinase. These data demonstrate that YopJ inhibits innate immune responses by acylating TAK1 (129). However, work on methylation or acylation influencing NF-κB signaling pathway is relatively limited, and further exploration is need to identify new methyltransferases or acetyltransferases involved in TAK1-TABs-mediated signaling, especially in mammal cells.

Like other post-translational modifications, sumoylation is a dynamic process that is mediated by E1, E2, and E3 enzymes, and has a pivotal role in a range of cellular functions (130). SUMO is an ubiquitin-like protein that binds to many target proteins in a covalently binding manner. Wang et al. have shown that TAB2 could be modified by SUMO at evolutionarily conserved Lys 329. In addition, PIAS3 is identified as a SUMO E3 ligase that interacts with and promotes sumoylation of TAB2. Interestingly, PIAS3 inhibits TAB2 activity in a dose-dependent manner, while blocking of sumoylation caused by Lys 329 mutation enhanced TAB2 activity (131). Although this study provides evidence supporting a role of sumoylation in the regulation of TAK1-TABs-mediated signaling, the examination of sumoylation *in vivo* and related mechanisms needs to been further investigated. In addition, it will be interesting to identify other new E3 ligases or desumoylating enzymes that are responsible for modulating sumoylation states of TAK1-TABs complex.

O-Linked β-N-acetylglucosamine (O-GlcNAc) is a ubiquitous dynamic post-translational modification known to target more than 3,000 eukaryotic proteins (132). O-GlcNAcylation is thought to regulate proteins in a manner similar to protein phosphorylation, and this modification regulates many cellular functions, such as cellular stress responses. TAB1 is modified at Ser395 by O-GlcNAcylation, which is important for autophosphorylation of TAK1 at Thr187 and activation of NF-κB signaling (132, 133). This is one of the first cases of a single O-GlcNAcylation site on a signaling protein that regulates key innate immune signaling pathways. After that, Tao et al. found that TAB3 is also modified by O-GlcNAcylation at the Ser408 position, which is necessary for TAK1 activation. Excessive O-GlcNAcylation modification of TAB3 will over-induce the activation of TAK1, which is related to tumor progression (134). Collectively, the regulation of O-GlcNAcylation of TAK1-TABs complex is important for immune and inflammatory responses and tumorigenesis.

## Conclusions and Perspectives

A series of knockout experiments proved that the TAK1-TABs complex is essential for IL-1R-, TNFR-, and TLRs-mediated signaling pathways that lead to activation of MAPKs and NF-κB (135). TAK1-TABs complex-mediated signaling is also correlated with a variety of diseases. TAK1-deficiency causes abnormal cell differentiation, increased cell death, and decreased inflammatory responses (49). In addition, TAK1-TABs is also associated with contact hypersensitivity (136) and neuronal death during cerebral ischemia (29). TAK1-TABs over-activation is related to the pathogenesis of autoimmune diseases and the development of cancer (49).

Although our understanding of the PTMs of TAK1-TABs has made significant progress, except for ubiquitination and phosphorylation, little is known about the functional role of other PTMs. And whether there are other forms of PTMs that have functional roles in regulating the TAK1-TABs complex and how these PTMs are related to each other is still unknown. In the future, we hope that more research will use high-resolution mass spectrometry measurement and advanced analysis technology platforms to perform more specific and sensitive detection of new protein changes of TAK1-TABs complex, and explore more detailed molecular mechanisms behind these modifications. These efforts will expand our understanding of inflammation and related cellular and molecular mechanisms, and provide new methods and strategies for effective control and treatment of inflammatory diseases.

## Author Contributions

C-QL conceived and designed this review. Y-RX and C-QL performed manuscript preparation, literature search, and editing. C-QL and Y-RX wrote the manuscript. All authors contributed to the article and approved the submitted version.

## Funding

The research in the authors’ laboratory was funded by the National Natural Science Foundation of China (31870870, 32070773), the Fundamental Research Funds for the Central Universities (2042019kf0204), the State Key Laboratory of Veterinary Etiological Biology Grant (SKLVEB2020KFKT002), the Wuhan University Experiment Technology Project Funding (WHU-2020-SYJS-05), and the Natural Science Foundation of Hubei Province (2018CFA016).

## Conflict of Interest

The authors declare that the research was conducted in the absence of any commercial or financial relationships that could be construed as a potential conflict of interest.
